# Population mixing and incidence of cancers in adolescents and young adults between 1990 and 2013 in Yorkshire, UK

**DOI:** 10.1007/s10552-016-0797-3

**Published:** 2016-08-12

**Authors:** A. Imam, L. Fairley, R. C. Parslow, R. G. Feltbower

**Affiliations:** 1 Department of Paediatrics, Aminu Kano Teaching Hospital, PMB 3452 Zaria road, Kano, Nigeria; 2 Division of Epidemiology and Biostatistics, School of Medicine, University of Leeds, Room 8.49, Worsley Building, Clarendon Way, Leeds, LS2 9JT UK

**Keywords:** Cancer, Teenagers and young adults, Population mixing

## Abstract

**Purpose:**

Epidemiological evidence suggests a role for an infectious etiology for cancers in teenagers and young adults (TYAs). We investigated this by describing associations between infection transmission using the population mixing (PM) proxy and incidence of cancers in TYAs in Yorkshire, UK.

**Methods:**

We extracted cancer cases from the Yorkshire Specialist Register of Cancer in Children and Young People from 1990 to 2013 (*n* = 1929). Using multivariable Poisson regression models (adjusting for effects of deprivation and population density), we investigated whether PM was associated with cancer incidence. We included population mixing–population density interaction terms to examine for differences in effects of PM in urban and rural populations.

**Results:**

Nonsignificant IRRs were observed for leukemias (IRR 1.20, 95% CI 0.91–1.59), lymphomas (IRR 1.09, 95% CI 0.90–1.32), central nervous system tumors (IRR 1.06, 95% CI 0.80–1.40) and germ cell tumors (IRR 1.14, 95% CI 0.92–1.41). The association between PM and cancer incidence did not vary in urban and rural areas.

**Conclusions:**

Study results suggest PM is not associated with incidence of cancers among TYAs. This effect does not differ between rural and urban settings.

**Electronic supplementary material:**

The online version of this article (doi:10.1007/s10552-016-0797-3) contains supplementary material, which is available to authorized users.

## Introduction

In the UK, cancer is the leading cause of death in teenage and young adult (TYA) populations between the ages of 15 and 24 years with very little known about its etiology [1]. Recent findings have, however, suggested infections might play a role in the etiology of cancers in this age-group [2]. This is because seasonality of tumor incidence has been described in relation to time of diagnosis and time of birth, and this might reflect a seasonal variation in infections [2].

Population mixing is seen as a proxy measure for infection transmission [3]. The original population mixing hypothesis proposed by Kinlen [4] suggests leukemia occurs as a rare response to a mini-epidemic arising from the intermixing of rural immunologically naive populations with migrants of predominantly urban origins. The hypothesis has been extended by other researchers to explain incidence of other cancers, particularly in children [5, 6].

Few studies have, however, investigated the effects of population mixing on the incidence of adolescent cancers [7]. An earlier study which examined associations between population mixing and incidence of leukemia, lymphoma and central nervous system tumors among 15–24 year olds diagnosed between 1996 and 2005 in England only found a significant inverse relationship for CNS tumors [7].

Our study aims to examine statistical associations between population mixing and incidence of cancers in TYAs aged between 15 and 24 years in Yorkshire, UK. In contrast to the earlier published study by van Laar et al., we considered an extended period of diagnosis between 1990 and 2013 and we determined whether any effects of population mixing differed among rural and urban population.

## Materials and methods

### Study population

Data on all individuals diagnosed with cancer between the ages of 15 and 24 years from 1990 to 2013 were extracted from the Yorkshire Specialist Register of Cancer in Children and Young People (YSRCCYP). The YSRCCYP is a population-based register which covers the Yorkshire and Humber Strategic Health Authority and has records of TYA cancer cases aged between 15 and 29 years dating back to 1990 [8].

Extracted data consisted of individual ages, sex, year of diagnosis, tumor diagnostic groups and postcodes (zip codes) at diagnosis which was mapped to an electoral ward based on the 1991 UK census. We also obtained population data based on 1991 census geography from the Office for National Statistics [9]. These included midyear populations by gender and 5-year age bands for all electoral wards in the Yorkshire region using 1991 UK census figures. We selected the 1991 census as our reference census because it is midway between the potential exposure window (1966–2013) for the effect of population mixing on the study population and thus might best reflect effects of population mixing on our study population. We also derived model covariates (Shannon index of diversity, Townsend deprivation index and person-weighted population density) for each electoral ward using data from the same reference census. The Shannon index is a measure of diversity and estimates levels of population mixing based on diversity of origins of incoming migrants into a defined area (electoral ward) from anywhere in England [5]. In-migrants are defined as the proportion of individuals with a different address in the year preceding the 1991 census and not those who merely moved within wards [5], nor does it take account of the distance moved by in-migrants. Higher values of this index suggest a greater diversity of in-migrants in the defined area. The Townsend deprivation index is an area-based measure of deprivation which uses readily available census data including proportion of unemployed persons, households not owner-occupied, overcrowded households and households without a car [10]. Person-weighted population density of an electoral ward is calculated by summing weighted averages of individual census enumeration districts within an electoral ward [11]. Previous research has identified both population density and deprivation to be confounding variables when analyzing effects of population mixing on incidence of cancers [5, 12].

We grouped our case data using the International Classification of Childhood Cancer (ICCC) coding [13] to classify tumor groups into 12 distinct categories. For the population mixing analysis, we, however, used four main tumor groups: leukemias, lymphomas and CNS tumors and germ cell tumors and further diagnostic subgroups for leukemia, including acute lymphoblastic leukemia (ALL) and acute myeloid leukemia (AML) and for lymphomas we included Hodgkin lymphoma (HL) and non-Hodgkin lymphoma (NHL). We, however, could not look at CNS tumor subgroups because of the small sample sizes of individual subgroups. These groups were included based on tumor groups and subgroups that have previously been examined by researchers focusing on the PM hypothesis, particularly in children [5–7]. These tumors have also been shown to demonstrate seasonality in incidence among TYAs [2]. We also included germ cell tumors as these are tumors are typical within this age range although no previous association with population mixing has been examined.

### Statistical analysis

We used Poisson or negative binomial models to observe for an association of incidence of tumors with population mixing. The negative binomial model was preferred if overdispersion was evident. Overdispersion was tested by running the negative binomial equivalent for the best fitting Poisson model. In cases where the *p* value of the likelihood statistic was <0.05, models were deemed to be overdispersed.

To derive an estimate of person-year which we used as our model offset term, we added population figures (derived from the 1991 census) for each electoral ward for 5-year age bands and sex for individuals aged between 15–19 year olds and 20–24 year olds and multiplied the total population for each ward by 24 (length of the study period). Model covariates included population mixing (measured using the Shannon index of diversity), person-weighted population density and deprivation measured using the Townsend score. These covariates were initially examined for collinearity. Person-based population density and Townsend index demonstrated collinearity (correlation coefficient of 0.77), so both variables were not included in the same model.

In our model building, we considered 2 initial univariable base models. A first model with population mixing as a continuous covariate and a second base model with tertiles of population mixing (model was divided into a low, medium and a high mixing category)—the latter to allow for any threshold effects associated with population mixing. In both models, we adjusted for age-group and sex. We then added categorical and continuous forms of population density and Townsend separately (but never in combination due to the collinearity) to the best fitting base model for each individual tumor group considered. A population mixing–population density interaction term was then added to each model to assess whether there was a significant improvement in fit. This was done to assess for differential effects of population mixing in a rural and an urban setting. Best fit univariable base models and multivariable models were all selected using Akaike’s information criteria (AIC) fit statistics, and all derived model coefficients were exponentiated to give IRRs and 95% confidence intervals. IRRs with corresponding 95% intervals were reported for each tumor group for univariable models which included population mixing as a continuous variable (this was the best fitting base model), multivariable models which involved adjustments for either Townsend index and population density as dictated by model fit statistics and a multivariable model which involved the addition of a population mixing–population density interaction to the best fitting multivariable model.

## Results

Between 1990 and 2013, there were 1,929 incident cases of cancer in individuals aged between 15 and 24 years, 61.7 % of whom were males and 38.3 % females. Table 1 shows the total number of incident cases divided into the main tumor groups with comparative proportions of each tumor across age-group and gender. The most common tumor groups overall were lymphomas (28.6 %), germ cell tumors (22.2 %), leukemias (13.4 %) and CNS tumors (13.2 %). Gender differences were observed in tumor incidence. Germ cell tumors were the most common in male and accounted for about a third of all such tumors. Lymphomas were the most common tumors in females, accounting for around a third of all tumors.

**Table 1:** Incident cases of tumors across gender and age-group

Tumor groups	Gender		Age-group		Total (%)
	Male (%)	Female (%)	15–19 (%)	20–24 (%)	
Leukemias	148 (12.4)	110 (14.9)	142 (17.0)	116 (10.6)	258 (13.4)
Lymphomas	298 (25.0)	254 (34.4)	236 (28.2)	316 (28.9)	552 (28.6)
CNS tumors	138 (11.6)	116 (15.7)	124 (14.8)	130 (11.9)	254 (13.2)
Germ cell tumors	385 (32.3)	43 (5.8)	141 (16.9)	287 (26.2)	428 (22.2)
Other solid tumors	221 (18.6)	216 (29.2)	192 (23.0)	245 (22.4)	437 (22.7)
Total	1190	739	835	1094	1929

Percentages are column percentages

Other solid tumor group includes neuroblastoma, renal tumors, hepatic tumors, malignant bone tumors soft tissue sarcoma, malignant epithelial neoplasms, other and unspecified malignant neoplasm

*CNS* central nervous system

Table 2 shows descriptive statistics for key variables. The Shannon index showed a small amount of variation between electoral wards (mean = 3.39, SD = 0.46).

**Table 2:** Summary table showing descriptive statistics of key exposure variables

Variable	Range	Median (IQR)	Mean (SD)
Shannon index	1.89 to 5.16	3.36 (3.09 to 3.68)	3.39 (0.5)
Townsend score	−4.8 to 17.9	−1.1 (−2.4 to 1.8)	0 (3.4)
Population density	0.01 to 51.0	5.5 (0.8 to 10.8)	7.0 (7.2)

Table 3 shows IRR and 95% confidence intervals for univariable models of population mixing, the best fitting multivariable model and a model containing the population mixing–population density interaction term (all models were adjusted for age and sex). The best fitting multivariable model involved adjusting for person-weighted population density score for most tumor groups and subgroups except CNS tumors, germ cell tumors and Hodgkin lymphoma for which an adjustment for the effect of Townsend deprivation resulted in the best fitting model. Most tumor groups and subgroups demonstrated a direct association between population mixing and risk of tumor incidence except NHL and AML subgroups which demonstrated an inverse relationship. These relationships were, however, not statistically significant for any diagnostic tumor group or subgroup. This level of association was evident for both univariable and multivariable models. Addition of an interaction term did not result in any distinct pattern of incidence of tumor groups in the tertiles of population density except for leukemias where there was a nonsignificant gradual increase in effect size from the first (lowest) tertile of population density to the third (highest) tertile.

**Table 3:** Incidence rate ratios (IRR), 95% confidence intervals models of population mixing, best fitting multivariable models and models with addition of a population mixing–population density interaction term

Diagnostic group	Population mixing	Age- and sex-adjusted model		Multivariable model*		Multivariable model with interaction term^#^		
		IRR	95% CI	IRR	95% CI	Tertiles of population density	IRR	95% CI
Leukemia	Continuous	1.19	0.90–1.56	1.20	0.91–1.59^a^	First tertile	1.02	0.44–2.36
						Second tertile	1.04	0.66–1.66
						Third tertile	1.49	0.98–2.28
Acute lymphoblastic leukemia (ALL)	Continuous	1.16	0.77–1.75	1.18	0.77–1.79^a^	First tertile	0.94	0.29–3.03
						Second tertile	1.27	0.62–2.62
						Third tertile	1.43	0.75–2.72
Acute myeloid leukemia (AML)	First tertile	1.0	–	1.0^a^		First tertile	1.34	0.41–4.39
	Second tertile	0.70	0.39–1.24	0.68	0.38–1.21	Second tertile	0.85	0.43–1.66
	Third tertile	1.09	0.66–1.78	1.08	0.66–1.77	Third tertile	1.39	0.72–2.69
Lymphoma	Continuous	1.09	0.90–1.32	1.09	0.90–1.32^a^	First tertile	0.69	0.34–1.39
						Second tertile	1.00	0.73–1.36
						Third tertile	1.22	0.91–1.63
Hodgkin lymphoma	Continuous	1.18	0.95–1.46	1.19	0.96–1.48^b^	First tertile	0.79	0.38–1.68
						Second tertile	1.08	0.75–1.54
						Third tertile	1.40	1.00–1.95
Non-Hodgkin lymphoma	Continuous	0.80	0.50–1.27	0.80	0.50–1.28^a^	First tertile	0.35	0.05–2.43
						Second tertile	0.94	0.45–1.95
						Third tertile	0.61	0.29–1.28
Central nervous system tumors	Continuous	1.09	0.83–1.44	1.06	0.80–1.40^b^	First tertile	1.10	0.54–2.22
						Second tertile	1.16	0.75–1.80
						Third tertile	1.04	0.65–1.66
Germ cell tumors	Continuous	1.15	0.93–1.43	1.14	0.92–1.41^b^	First tertile	0.99	0.55–1.77
						Second tertile	1.54	1.09–2.19
						Third tertile	0.99	0.71–1.38

* Multivariable models are best fit multivariable models and do not have a population mixing–population density interaction term added, age and sex adjusted

^a^Model estimates are adjusted for population density as a continuous variable

^b^Model estimate is adjusted for Townsend score as a continuous variable

^#^Interaction term is a population mixing–population density interaction term which was added to the best fit multivariable model containing population density as a covariate. The first tertile represents areas with the lowest third of population densities while the third tertile represents areas with the highest third of population densities

## Discussion

Our study investigated whether there was any evidence of a relationship between population mixing and cancers occurring in TYAs. We found no significant association between population mixing and incidence of leukemias, lymphomas, CNS tumors and germ cell tumors occurring in TYAs. The addition of a population mixing–population density interaction term was not significant across tertiles of population density. Tertiles of population density were used as a proxy to determine whether wards were rural or urban with wards in the lowest tertiles representing more rural wards, while those in the highest tertile represented more urban wards. Our results therefore indicate that the level of rurality did not affect the observed association with population mixing.

The findings of a nonsignificant association between population mixing and incidence of tumors in TYAs contrast with Kinlen’s population mixing hypothesis which describes a direct association between childhood leukemia and population mixing [4]. Kinlen proposes that childhood leukemia is an uncommon response of an immunologically naive rural and geographically isolated population exposed to an otherwise commonplace infection due to a sudden influx of a predominantly urban population [4]. Although Kinlen’s original hypothesis was restricted to leukemia and its occurrence in childhood, the concept of population mixing has, however, been extended by researchers beyond this specific hypothesis and has been used as a proxy measure for infection spread among populations [3]. Researchers have thus examined relationships between incidence of cancers and population mixing when a biological plausibility for an infectious cause for cancer exists. Our study findings also contrast with the Greaves immunological model [14]. In this model, Greaves hypothesizes that childhood leukemia arises from immune dysregulation occurring as a result of a delayed exposure to infection in infancy, thus suggesting early life exposures to infection might protect against childhood leukemia.

Our study findings are similar to two previous studies conducted in children. Parslow et al. [5] in the UK in 2002 also demonstrated nonsignificant associations for CNS tumors, while Dockerty et al. [15] in a study conducted in rural New Zealand in 1996 demonstrated nonsignificant associations for childhood leukemia. The only other study that has examined effects of population mixing exclusively in the TYA group is a recent study by van Laar et al. [7]. This study described an inverse association between incidence of CNS tumors and population mixing in TYAs. A possible explanation for this difference might be geographical since van Laar et al. considered the effect of population mixing in the whole of UK, whereas this study was limited to the Yorkshire region. Because population mixing is a proxy for infection transmission, it is possible that the putative agent associated with incidence of CNS tumors in these age-groups might not be widely distributed in the population and thus might not have been present in the Yorkshire region. This might have implications in a study investigating the effect of population mixing on the incidence of CNS tumors as study area size might affect results. Future research investigating this might also highlight possible differences. However, our study differed from the study by van Laar et al. by (1) extending the period of analysis to 1990–2013 from 1996 to 2005 and (2) exploring the effect of interaction terms on population mixing. We have, however, used a smaller study population than van Laar’s study which looked at TYAs in the whole of England and so we were unable to perform subgroup analyses for CNS tumors due to small numbers of cases.

We also included germ cell tumors in our analysis as this group represents the second most frequent diagnosis within the TYA age range. There is no previous evidence to suggest an association between population mixing and germ cell tumors, and we did not find a statistically significant association.

Our study findings, however, contrast with earlier works by Kinlen et al. [16] and Clark et al. [17]. Although these studies were carried out in childhood populations rather than TYAs, other reasons might exist for differences between our study findings and these studies. One reason for these differences might be explained by different approaches to study the effect of mixing. While Kinlen et al. and Clark et al. have derived estimates of relative risks by dividing observed case counts by expected case counts derived from standardized incidence rates (SIRs), this study has used regression analysis. Studies using rates to determine effects of population mixing might be quite sensitive to slight changes in either the numerator or denominator. In instances where even a few observed cases were missed, estimates of relative risks would tend to be markedly lower than the true effect size; the converse of an erroneous exaggerated relative risk might apply if observed cases were overrepresented. Future research replicating our study using SIRs might highlight how effect estimates could differ when varying methods are used in an analysis of population mixing.

## Strengths and limitations

Our study is one of the few studies that have described the effects of population mixing in the TYA populations. We have also adjusted for the confounding effects of population density in the interrelationship between population mixing and incidence of cancers using person-weighted population densities. Such weighted densities have been shown to be a better and more accurate reflection of population density than area-based densities [18]. Using such estimates should lead to improved accuracy of our effect estimates.

We have also geo-coded all potential study subjects from the population-based specialist cancer registry to an electoral ward of diagnosis; thus, because of this and the high levels of case ascertainment [19], selection bias is likely to be minimal.

Comparatively, the Yorkshire region might have a smaller population and thus smaller potential study participants than most studies conducted in entire countries or regions with a larger population. We, however, attempted to address that deficiency by considering a longer study period of 24 years, thus accruing a larger sample of potential study subjects. Although this helped with most of our analyses, our ability to perform subgroup analysis, in particular for CNS subgroups, was limited. Our study design was ecological, so it may be prone to the ecologic fallacy and so findings from this study cannot be ascribed to individuals within the wards. Population denominators for offset terms in the population mixing models have also been multiplied by the length of study period to derive the person-years offset; this is based on an assumption that population denominators did not change much during the study period. If an electoral ward experienced a significant net increase in population during the study period, population denominators would have been underestimated leading to exaggerated effect estimates. The converse also applies for a net decrease in population. Reviewing the population change in the Yorkshire region from ONS statistics [8] suggested a 2 % decrease in population of 15–24 year olds between 1990 and 2013, suggesting the population denominator might not have changed significantly during the study period.

## Conclusions

We did not find a statistically significant relationship between population mixing and incidence of leukemia, lymphoma, CNS tumors or germ cell tumors for TYAs in Yorkshire. Although a previous study had described a relationship between CNS tumors and population mixing in this age-group, further analyses investigating what effects geography might play in these differences would be valuable.

## Electronic supplementary material

Below is the link to the electronic supplementary material.

Supplementary material 1 (DOCX 17 kb)
