# Supplementary material for: Population mixing and incidence of cancers in adolescents and young adults between 1990 and 2013 in Yorkshire, UK
Source: Cancer Causes Control. 2016 Aug 12;27(10):1287–92. doi: 10.1007/s10552-016-0797-3 (PMC5025504; doi:10.1007/s10552-016-0797-3)
Supplement: Supplementary file 1 — Supplementary material 1 (DOCX 17 kb) [file 10552_2016_797_MOESM1_ESM.docx]

**Population mixing and incidence of cancers in adolescents and young adults between 1990 and 2013 in Yorkshire, UK**

A Imam, L Fairley, R.C. Parslow, R.G. Feltbower

**Supplementary Table**

**Supplementary Table 1 - Akaike’s information criteria (AIC) fit statistics for all models used to determine the best fitting model for main tumour groups and subgroups in population mixing analysis**

| **Model** | **Variables** | **Leukaemia** | **Acute lymphoblastic leukaemia** | **Acute myeloid leukaemia**** | **Lymphoma** | **Hodgkin lymphoma** | **Non-Hodgkin lymphoma** | **CNS tumors** | **Germ cell tumors** |
| --- | --- | --- | --- | --- | --- | --- | --- | --- | --- |
| Model 1 | Population mixing (Continuous) | 1417.5 | 767.6* | 815.1 | 2307.5* | 1929.8 | 705.0* | 1444.2 | 1740.2* |
| Model 2 | Population mixing (Categorical) | 1419.9 | 768.3 | 813.4* | 2310.2 | 1933.6 | 705.3 | 1446.3 | 1741.4 |
| Model 3 | Population mixing +Population density(continuous) | 1415.1* | 767.9 | 814.8 | 2308.1 | 1930.7 | 706.3 | 1445.8 | 1742.1 |
| Model 4 | Population mixing +Population density(categorical) | 1420.2 | 769.4 | 817.4 | 2308.1 | 1932.7 | 706.9 | 1444.7 | 1743.7 |
| Model 5 | Population mixing +deprivation (continuous) | 1417.1 | 768.1 | 815.4 | 2308.5 | 1929.6* | 707.0 | 1441.3* | 1741.2 |
| Model 6 | Population mixing +deprivation (categorical) | 1419.8 | 768.8 | 816.8 | 2311.1 | 1933 | 708.4 | 1444.6 | 1743.7 |
| Model 7 | Population mixing +Population density(categorical) + interaction term | 1422.7 | 773.8 | 821.9 | 2309.5 | 1934.3 | 709.6 | 1448.6 | 1744.0 |

All models age and sex adjusted

*****Best fit model

** AML model population mixing categorical for models 3 - 6
